# Reclassification of type strains of Rhizobium indigoferae and Sinorhizobium kummerowiae into the species Rhizobium leguminosarum and Sinorhizobium meliloti, respectively

**DOI:** 10.1099/ijsem.0.006451

**Published:** 2024-07-22

**Authors:** Esther Menéndez, José David Flores-Félix, Sabhjeet Kaur, George C. diCenzo, J. Peter W. Young, Alvaro Peix, Encarna Velázquez

**Affiliations:** 1 Departamento de Microbiología y Genética, Universidad de Salamanca, Salamanca, Spain; 2 Instituto de Investigación en Agrobiotecnología (CIALE), Universidad de Salamanca, Salamanca, Spain; 3 Grupo de Interacción Planta-Microorganismo, USAL, Unidad Asociada al CSIC por el IRNASA, Salamanca, Spain; 4 Department of Biology, Queen’s University, Kingston, Ontario, Canada; 5 Department of Biology, University of York, York YO10 5DD, UK; 6 Instituto de Recursos Naturales y Agrobiología, IRNASA-CSIC, Salamanca, Spain

**Keywords:** phylogeny, reclassification, *Rhizobium indigoferae*, *Rhizobium leguminosarum*, *Rhizobiaceae*, *Sinorhizobium kummerowiae*, *Sinorhizobium meliloti*

## Abstract

The species *Rhizobium indigoferae* and *Sinorhizobium kummerowiae* were isolated from legume nodules and the 16S rRNA sequences of their respective type strains, CCBAU 71042^T^ and CCBAU 71714^T^, were highly divergent from those of the other species of the genera *Rhizobium* and *Sinorhizobium*, respectively. However, the 16S rRNA gene sequences obtained for strains CCBAU 71042^T^ and CCBAU 71714^T^ several years after description, were different from the original ones, showing 100 % similarity to the type strains of *Rhizobium leguminosarum* and *Sinorhizobium meliloti*, respectively. Phylogenetic analyses of two housekeeping genes, *recA* and *atpD*, confirmed the high phylogenetic closeness of strains CCBAU 71042^T^ and CCBAU 71714^T^ to the respective type strains of *R. leguminosarum* and *S. meliloti*. In the present work, we compared the genomes of the type strains of *R. indigoferae* and *S. kummerowiae* available in several culture collections with those of the respective type strains of *R. leguminosarum* and *S. meliloti*, some of them obtained in this study. The calculated average nucleotide identity–blast and digital DNA–DNA hybridization values in both cases were higher than those recommended for species differentiation, supporting the proposal for the reclassification of the type strains of *R. indigoferae* and *S. kummerowiae* into the species *R. leguminosarum* and *S. meliloti*, respectively.

The species *Rhizobium indigoferae* and *Sinorhizobium kummerowiae* were isolated from nodules of the legumes *Indigofera* and *Kummerowia* in China [1]. They were described in the *International Journal of Systematic and Evolutionary Microbiology* in 2002, but as their type strains were initially deposited in a single recognized culture collection as *R. indigoferae* CCBAU 71714^T^ and *S. kummerowiae* CCBAU 71042^T^, both names were considered not validly published at the time of the original publication, and only after a second deposit in 2003 in the Institut Pasteur Collection (CIP) as *R. indigoferae* CIP 108029^T^ and *S. kummerowiae* CIP 108026^T^ were the names considered valid by the Judicial Commission [2]. From 2003 onwards, the type strains of both species were deposited in other culture collections according to the information recorded in BacDive webpage (https://bacdive.dsmz.de/).

The proposal of the species *R. indigoferae* and *S. kummerowiae* was based on a polyphasic approach, including an analysis of the 16S rRNA gene sequences of their type strains deposited in the Culture Collection of Beijing Agricultural University (CCBAU), which were divergent from those of the other species of their respective genera. The sequence deposited under the accession AF364068 from the CCBAU type strain of *R. indigoferae* was related to species belonging to the phylogenetic group of *Rhizobium gallicum*, while that corresponding to AF364067 from the CCBAU type strain of *S. kummerowiae* was related to species belonging to the phylogenetic group of *S. fredii* [1].

In the abstracts of the descriptions of *R. indigoferae* and *S. kummerowiae*, these type strains were erroneously cited as *R. indigoferae* CCBAU 71714^T^ (=AS 1.3046) and *S. kummerowiae* CCBAU 71042^T^ (AS 1.3045) [3]. These errors were later corrected and currently the List of Prokaryotic names with Standing in Nomenclature indicates that strains CCBAU 71042^T^ (16S rRNA gene sequence accession AF364068) and CCBAU 71714^T^ (16S rRNA gene sequence accession AF364067) are the type strains of *R. indigoferae* and *S. kummerowiae*, respectively. The 16S rRNA gene sequences of both strains were deposited twice into GenBank in 2001, using the accession numbers AF364068.1 and AY034027.1 for CCBAU 71042^T^, and the accession numbers AY034028.1 and AF364067.1 for CCBAU 71714^T^. Two of these accessions were subsequently updated twice in 2008 reaching version 3, which replaced versions 1 and 2, namely AY034027.3 for *R. indigoferae* CCBAU 71042^T^ and AF364067.3 for *S. kummerowiae* CCBAU 71714^T^. In the case of the species *R. indigoferae*, the 16S rRNA sequence of the type strain NBRC 100398^T^ was deposited in GenBank in 2011 (accession AB681167) and in 2020 that of *R. indigoferae* CCBAU 71042^T^ was deposited again in GenBank (accession MT062523). These two sequences were identical to those updated in 2001 and 2008 (Fig. 1).

**Fig. 1.**
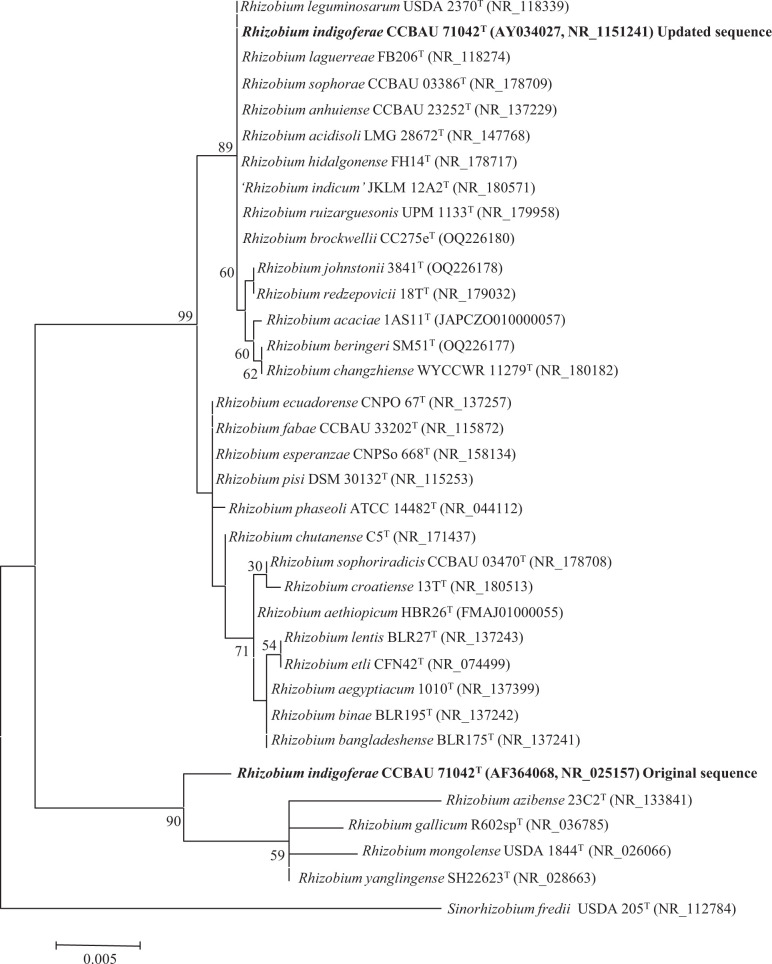
Maximum-likelihood phylogenetic tree based on 16S rRNA gene sequences (1215 nt) including the original and the updated sequences of *R. indigoferae* CCBAU 71042^T^ within the genus *Rhizobium*. Bootstrap values calculated from 1000 replicates are indicated. Bar, 0.5 nt substitution per 100 nt. The sequences from accessions MT062523 (*R. indigoferae* CCBAU 71042^T^) and AB681167 (*R. indigoferae* NBRC 100398^T^) were identical to that from the accession AY034027 of *R. indigoferae* CCBAU 71042^T^.

Here, we used both GenBank accessions to perform a phylogenetic analysis using the ClustalW program for gene alignment [4] and the mega 7.0 software package [5] to build phylogenetic trees with the maximum-likelihood (ML) method [6]. Kimura’s two-parameter model [7] was used for the ML phylogeny. The results showed that the updated sequences of *R. indigoferae* CCBAU 71042^T^ (accession AY034027.3=NR_115124.1) and *S. kummerowiae* CCBAU 71714^T^ (accession AF364067.3=NR_114614.1) are phylogenetically divergent from the original sequences (Figs1  2). Notably, the updated sequences differ in 30 and 15 nucleotides, respectively, from those used for the proposal of the description of the species *R. indigoferae* and *S. kummerowiae* and they are instead identical to those of the type strains of several species of the *R. leguminosarum* group and to that of the type strain of *S. meliloti*, respectively (Figs1  2).

**Fig. 2.**
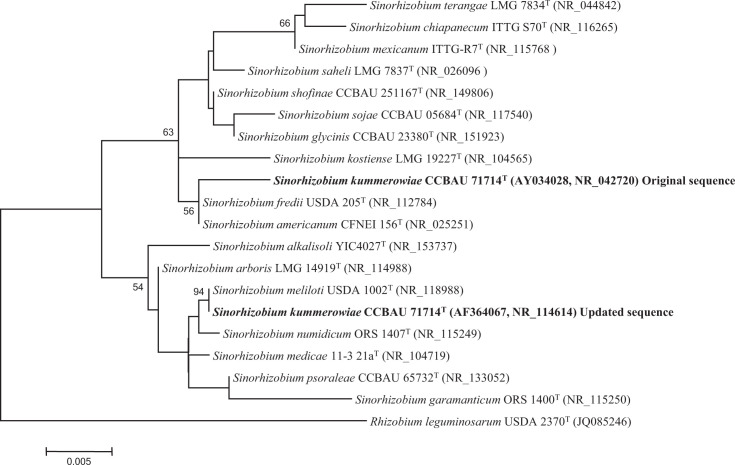
Maximum-likelihood phylogenetic tree based on 16S rRNA gene sequences (1308 nt) including the original and the updated sequences of *S. kummerowiae* CCBAU 71714^T^ within the genus *Sinorhizobium*. Bootstrap values calculated from 1000 replicates are indicated. Bar, 0.5 nt substitution per 100 nt.

Traditionally, the analysis of two housekeeping genes, *recA* and *atpD*, has been used to differentiate very closely related species in the genera *Rhizobium* and *Sinorhizobium* [8], as even 100 % identity in the 16S rRNA gene sequences between rhizobial strains does not imply that they belong to the same species. Therefore, we performed phylogenetic analyses using the *R. indigoferae* CCBAU 71042^T^ and *S. kummerowiae* CCBAU 71714^T^ *recA* and *atpD* gene sequences deposited in GenBank in 2006. Analysis of these concatenated genes using the same methodologies used for the 16S rRNA gene analysis further indicated that *R. indigoferae* CCBAU 71042^T^ and *S. kummerowiae* CCBAU 71714^T^ are phylogenetically very close to the type strains of *R. leguminosarum* and *S. meliloti*, respectively, suggesting that they belong to these species (Figs3  4).

**Fig. 3.**
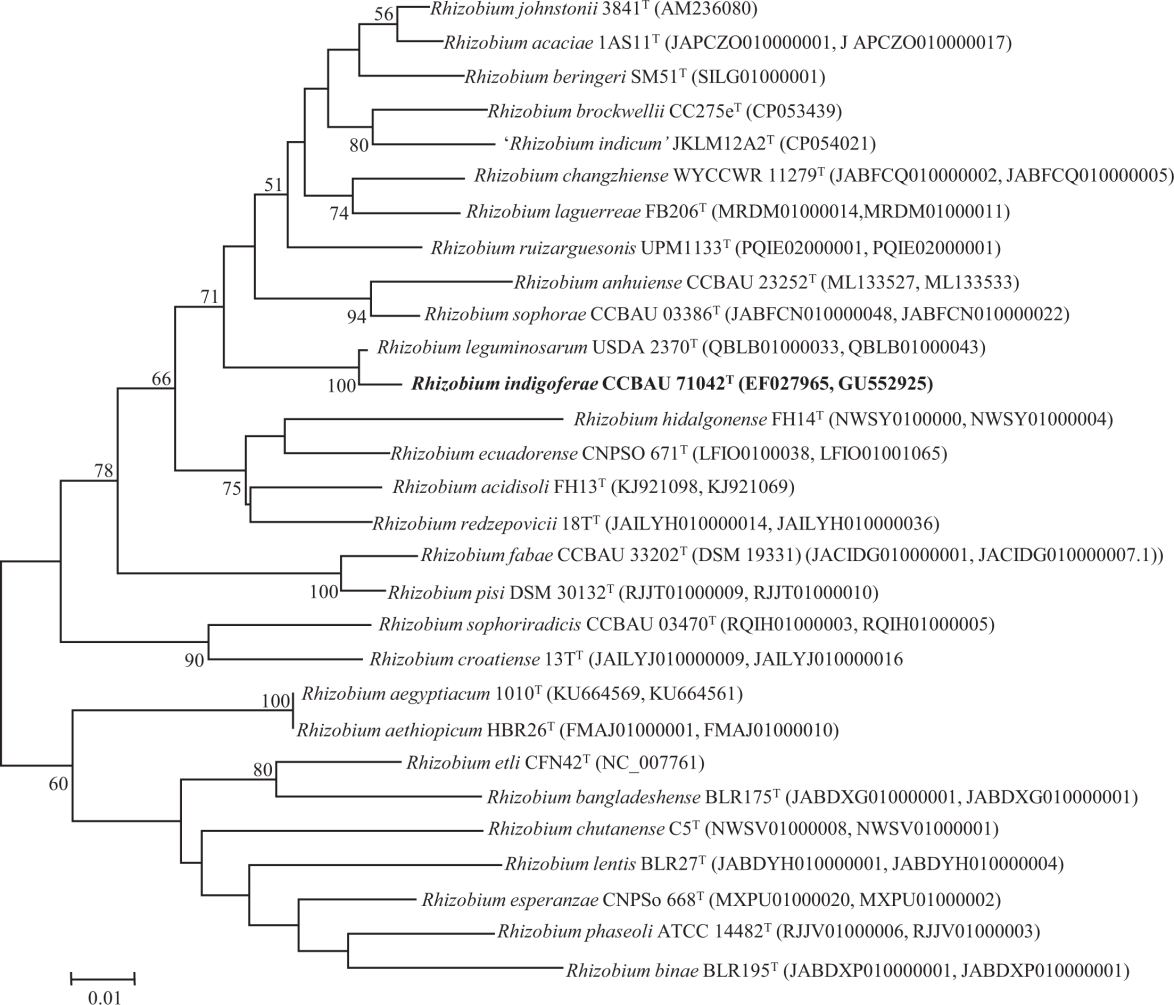
Maximum-likelihood phylogenetic tree based on concatenated *recA* and *atpD* genes (757 nt) showing the position of *R. indigoferae* CCBAU 71042^T^ within the genus *Rhizobium*. Bootstrap values calculated from 1000 replicates are indicated. Bar, 1 nt substitution per 100 nt.

**Fig. 4.**
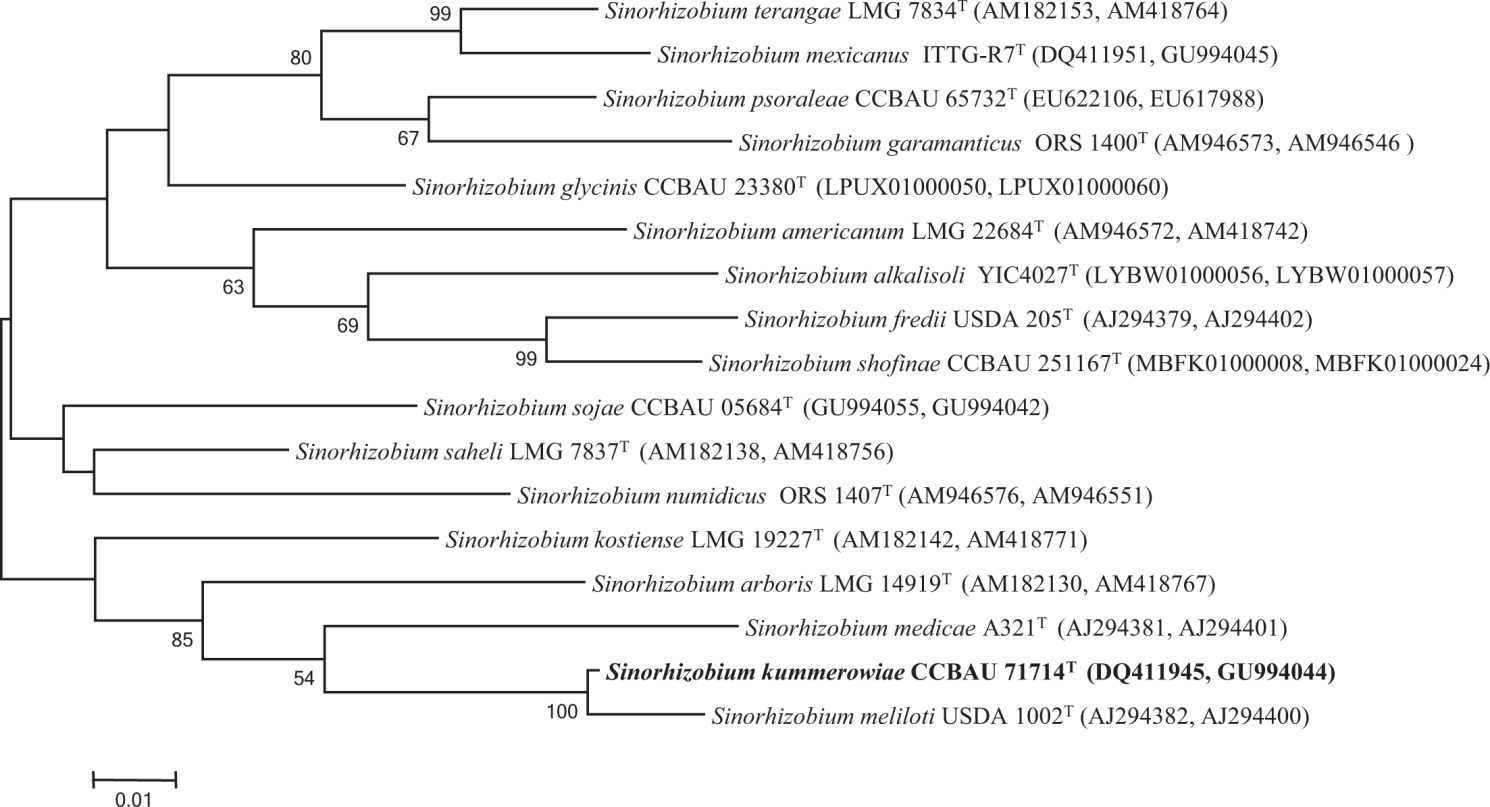
Maximum-likelihood phylogenetic tree based on concatenated *recA* and *atpD* genes (785 nt) showing the position of *S. kummerowiae* CCBAU 71714^T^ within the genus *Sinorhizobium*. Bootstrap values calculated from 1000 replicates are indicated. Bar, 1 nt substitution per 100 nt.

The genomes of *R. indigoferae* CIP 108029^T^ and *S. kummerowiae* CCBAU 71714^T^ and CIP 108026^T^ were obtained in this study (Table S1, available in the online Supplementary Material) using a previously described methodology [9], while those of *R. indigoferae* CCBAU 71042^T^ and NBRC 100398^T^ were retrieved from GenBank. Overall genomic relatedness indexes between the type strains of *R. leguminosarum* and *R. indigoferae* and between those of *S. meliloti* and *S. kummerowiae* were calculated. Average nucleotide identity–blast (ANIb) was calculated using the JSpecies server [10,11], while digital DNA–DNA hybridization (dDDH) values were calculated using the Genome-to-Genome Distance Calculator version 2.1 from DSMZ [12,13]. The calculated ANIb and dDDH values among *R. indigoferae* CCBAU 71042^T^, CIP 108029^T^ and NBRC 100398^T^ and between *S. kummerowiae* CCBAU 71714^T^ and CIP 108026^T^ were nearly 100 % in both cases. ANIb and dDDH values between *R. leguminosarum* USDA 2370^T^ and the type strains of *R. indigoferae* were higher than 97 and 81 %, respectively (Table S2). Similarly, the ANIb and dDDH values between *S. meliloti* USDA 1002^T^ and the type strains of *S. kummerowiae* were higher than 98 and 86 %, respectively (Table S2). These values are higher than those proposed for species differentiation [14] and confirm unambiguously that strains CCBAU 71042^T^ (=CIP 108029^T^=NBRC 100398^T^) and CCBAU 71714^T^ (=CIP 108026^T^) belong to the species *R. leguminosarum* and *S. meliloti*, respectively. These results disagree with those obtained by Wei *et al*. [1] using conventional DDH, which in agreement with their original 16S rRNA gene sequencing, showed low DNA–DNA relatedness values (less than 40 % in both cases) between the original type strains *R. indigoferae* CCBAU 71042^T^ and *S. kummerowiae* CCBAU 71714^T^ with respect to the type strains of their respective most closely related species, *R. leguminosarum* USDA 2370^T^ and *S. meliloti* USDA 1002^T^.

Based on these results, we propose the reclassification of the currently available strains CCBAU 71042^T^ (=CIP 108029^T^=NBRC 100398^T^) and CCBAU 71714^T^ (=CIP 108026^T^) into the species *R. leguminosarum* and *S. meliloti*, respectively. Therefore, a search must be made for suitable replacements of these type strains that meet the original characteristics described by Wei *et al*. [1], or neotypes should be designated according to Rule 18c, since both species contained several strains in addition to the type ones [1]. Only in the case that the original type strains or neotypes cannot be found, will the taxonomic status of the species *R. indigoferae* and *S. kummerowiae* have to be revised.

## supplementary material

10.1099/ijsem.0.006451 Uncited Table S1.

## Abbreviations

ANI: average nucleotide identity
dDDH: digital DNA–DNA hybridization
ML: maximum-likelihood
