# Supplementary material for: Reclassification of type strains of Rhizobium indigoferae and Sinorhizobium kummerowiae into the species Rhizobium leguminosarum and Sinorhizobium meliloti, respectively
Source: Int J Syst Evol Microbiol. 2024 Jul 22;74(7):006451. doi: 10.1099/ijsem.0.006451 (PMC11316585; doi:10.1099/ijsem.0.006451)
Supplement: Uncited Table S1. [file ijsem-74-06451-s001.pdf]

INTERNATIONAL JOURNAL OF SYSTEMATIC AND EVOLUTIONARY MICROBIOLOGY

SUPPLEMENTARY MATERIAL

Short communication

Reclassification of type strains of *Rhizobium indigoferae* and *Sinorhizobium kummerowiae* into the species *Rhizobium leguminosarum* and *Sinorhizobium meliloti*, respectively.

Esther Menéndez[1,2], José David Flores-Félix[1], Sabhjeet Kaur[3], George C diCenzo[3], J. Peter W. Young[4], Alvaro Peix[5,6]*, Encarna Velázquez[1,2,6]

1. Departamento de Microbiología y Genética, Universidad de Salamanca, Salamanca, Spain

2. Instituto de Investigación en Agrobiotecnología (CIALE), Universidad de Salamanca, Salamanca, Spain

3. Department of Biology, Queen's University, Kingston, Ontario, Canada

4. Department of Biology, University of York, York YO10 5DD, UK

5. Instituto de Recursos Naturales y Agrobiología, IRNASA-CSIC, Salamanca, Spain

6. Grupo de Interacción Planta-Microorganismo, USAL, Unidad Asociada al CSIC por el IRNASA, Salamanca, Spain.

Corresponding Author: Alvaro Peix. Instituto de Recursos Naturales y Agrobiología, IRNASA-CSIC, C/Cordel de Merinas 40-52, 37008 Salamanca, Spain. E-mail: alvaro.peix@csic.es

Table S1. Genome properties of the strains analysed in this study. Strains: 1, *R. leguminosarum* USDA 2370^{T} (QBLB00000000); 2, *R. indigoferae* CCBAU 71042^{T} (JABFCO010000000); 3, *R. indigoferae* NBRC 100398^{T} (BSOQ00000000); 4, *R. indigoferae* CIP 108029^{T} (CP140635-CP140639); 5, *S. meliloti* USDA 1002^{T} (JAGIOS000000000); 6, *S. kummerowiae* CCBAU 71042^{T} (CP120364-CP120366); 7, *S. kummerowiae* CIP 108026^{T} (CP140299-CP140301).

	**1**	**2**	**3**	**4**	**5**	**6**	**7**
BioProject	PRJNA450070	PRJNA611645	PRJDB10359	PRJNA1050618	PRJNA696473	PRJNA935862	PRJNA935862
BioSample	SAMN08929321	SAMN14341972	SAMD00238708	SAMN38748742	SAMN17673269	SAMN33342371	SAMN38748741
GenBank Accessions	QBLB00000000	JABFCO010000000	BSOQ00000000	CP140635-CP140639	JAGIOS000000000	CP120364-CP120366	CP140299-140301
Assembly	GCF_003058385.1	GCF_013087605.1	GCF_030160595.1	GCF_034554815.1	GCF_017876815.1	GCF_030064595.1	GCF_035326845.1
Size (Mb)	7,851,935	7,498,842	7,572,363	7,640,287	7,343,325	6,596,872	6,711,434
Contigs	108	91	81	5	4	3	3
GC%	60.6	60.7	60.7	60.6	62.0	62.1	62.1
Genes (total)	7,623	7,328	7,384	7,410	7,078	6,268	6,402
Genes (coding regions, proteins)	7,246	6,984	7,044	7,045	6,612	5,947	6,038
Pseudogenes	323	292	288	299	398	254	296
RNAs (rRNA, tRNA, other RNA)	54 (4, 46, 4)	52 (2, 46, 4)	52 (2, 46, 4)	66 (9, 53, 4)	68 (9, 55, 4)	67 (9, 54, 4)	68 (9, 55, 4)
N50	413,138	304,730	364,412	4,863,510	3,905,231	3,634,833	3,634,832
L50	5	9	8	1	1	1	1

Table S2. Average values in percentage of ANIb (below the diagonal) and dDDH (above the diagonal). Strains: 1, *Rhizobium leguminosarum* USDA 2370^T (QBLB00000000); 2, *Rhizobium indigoferae* CCBAU 71042^T (JABFCO010000000); 3, CIP 108029^T (CP140635-CP140639); 4, NBRC 100398^T (BSOQ00000000); 5, *Sinorhizobium meliloti* USDA 1002^T (JAGIOS000000000); 6, *Sinorhizobium kummerowiae* CCBAU 71714^T (CP120364-CP120366); 7, CIP 108026^T (CP140299-CP140301)

	1	2	3	4	5	6	7
1	*	81.50	81.40	81.40			
2	97.17	*	99.90	100			
3	97.12	99.88	*	100			
4	97.10	99.91	100	*			
5					*	87.00	86.60
6					98.20	*	100
7					98.09	99.92	*
